# Accidental paradiplomats? The curious case of Ontario school board budgets and Canadian soft power projection

**DOI:** 10.1177/00207020241232989

**Published:** 2024-02-22

**Authors:** Michael P.A. Murphy

**Affiliations:** Department of Political Studies, 4257 Queen’s University, Kingston, Ontario, Canada

**Keywords:** soft power, Canadian foreign policy, International Relations theory, international relations, international education, school boards, education policy, Ontario, education funding, student exchanges

## Abstract

From the earliest studies of soft power in International Relations, the importance of educational exchanges has been well-established. Studies of international education in the context of Canadian soft power often draw on cases from the higher education sector. This article argues that greater attention should be paid to the K-12 level, especially as budgetary pressures in Ontario’s education system are leading school boards to rapidly expand their international student recruitment efforts. Although this is not an example of intentional soft power projection, it nevertheless represents an important reminder that subnational actors may accidentally become paradiplomats whose actions have consequences on the international level. Further, this case reveals the importance of paying attention to actors typically overlooked by IR scholarship. Drawing on Joseph Nye’s theory of soft power and in conversation with prior research on international education as a mechanism of soft power projection, this article traces the thread between budgetary pressures in Ontario school boards and the broader context of soft power projection.

## Introduction

In *Soft Power: The Means to Success in World Politics,* Joseph Nye notes that one of the difficult elements of managing soft power is that its sources are not centralized.^1^ Instead, soft power emerges from multiple sources. This decentralization stands in stark contrast to hard power, the coercive capacity of a nation that is much more frequently understood as residing in the hands of national government decision-makers. Soft power is an alternative operation for influence that sees the preferences of one state align with another not by force, threat, or promise, but because preferences are shifted into greater coherence. This shift takes place largely within the domain of culture.

The role of education in shaping culture and society is well-established in the literature on the sociology of education,^2^ and it is little surprise that theorists of soft power would identify the strategic importance of student exchanges in projecting international power.^3^ Nye points to the liberalizing influence that students from the Soviet Union who participated in student exchanges throughout the Cold War would go on to have during their careers. While other academic exchange programs have received attention in recent years due to allegations of espionage to benefit of China and the impact of international students at the postsecondary level on the Canadian housing market, the same level of attention has not been paid to the impact of primary and secondary education exchanges (especially from the perspective of international politics).

In this article I explore recent changes to Canadian soft power projection through K-12 student exchanges. Such exchanges have flown largely below the radar of IR scholars, due in large part to Ontario school boards being *accidental *paradiplomats. Indeed, school boards in Ontario are a curious level of government. Often, school boards receive little public engagement and experience low voter turnout in elections, and elected trustees serve part-time with low remuneration while being expected to offer a publicly-accountable balance to full-time professional administrators. Corporate governance models limit the political power of trustees and school boards, and the replacement of local taxation powers with centralized per-pupil funding from the Ministry of Education has reduced their ability to set their own fiscal priorities.^4^ These constraints to school board agency are significant; by calling attention to Ontario school boards I do not wish to downplay these limitations. My argument is that we must pay attention to Ontario school boards—despite their limitations of agency—if we want to understand Canadian soft power projection today, because the boards have accidentally become paradiplomatic agents of Canadian soft power projection.

Scholars of Canadian political science and IR have explored paradiplomacy from multiple perspectives. In contrast to track two or unofficial diplomacy, paradiplomacy sees actors functioning in official capacities, but representing subnational, rather than national, units.^5^ Perhaps the most familiar case of paradiplomacy in Canada has been the Québécois efforts to project identity, increase international engagements with the broader Francophone world, and, at times, strengthen claims to sovereignty.^6^ Other provinces, some cities, and even non-governmental actors such as police departments have undertaken paradiplomacy,^7^ although not at the scale of the provincial government of Quebec. As Leah Sarson has recently argued, Indigenous governance can also be understood from the perspective of paradiplomacy, given the influence that Indigenous nations have had on international relations.^8^ Considered as an instance of both soft power projection and paradiplomacy, the impacts of the growth of student exchanges in response to Ontario school board budget pressures is doubly curious. Not only do we have a limited-agency actor engaging at a higher level of analysis than may be typical for that actor, but we also have an example of how paradiplomacy can arise accidentally. This case study merits further attention from IR scholars in Canada as well as from political scientists and policy analysts examining the business of school board governance. The first section explores the concept of soft power and its relationship to educational programming. The second section turns to the case study of Ontario school board budgetary pressures to provide context for the growth of student exchange strategies, while the third explores the implications of Ontario school boards as accidental paradiplomats of soft power projection. The conclusion reflects on future research trajectories and the complex role of school boards in Canadian governance.

## Education and soft power projection

The concept of soft power arose in the twilight of the Cold War, as IR theorists sought to make sense of a world that was shifting away from coercive power politics towards more subtle forms of influence. In response to trends of hard power's reduced fungibility, tangibility, and coerciveness, Joseph Nye argued that “soft power resources” were becoming more important for the successful navigation of world politics, placing at centre stage the global reach of American culture in attracting others to shared priorities.^9^ Rather than influencing the particular outcome of a decision through direct incentives or threats, soft power works as an *ex ante* influence to set the terms of one nation’s decision in a manner favourable to the nation exercising influence via soft power. Nye states that “soft power rests on the ability to set the political agenda in a way that shapes the preferences of others,” demonstrating the foundational logic that “if I can get you to *want* to do what I want, then I do not have to force you to do what you do *not* want to do.”^10^ Stated in such terms, it seems almost self-evident that a soft power strategy would reduce costs, risks, and uncertainties by bringing another’s preferences in line with one’s own. The question, of course, is how this can be accomplished. This section reviews the concept of soft power, focusing particularly on Nye’s original formulation to demonstrate the longstanding connection between education and soft power.^11^

Nye would go into much greater detail in the definition of soft power in his 2004 book of that title, exploring not only the nature but also the sources of soft power. The conceptualization of soft power as an influence on the preferences of others remains constant; however, the sustained reflection permits greater nuance, especially as it pertains to the decentralization of soft power. In an introductory reflection, Nye draws a parallel to a familiar concept from political economy that illustrates the concept while also indicating the difficulty of wielding soft power with the same dexterity as was possible with traditional forms of hard power:

Much as Adam Smith observed that people are led by an invisible hand when making decisions in a free market, our decisions in the marketplace for ideas are often shaped by soft power—an intangible attraction that persuades us to go along with others’ purposes without any explicit threat or exchange taking place.^12^

The invisibility of the hand here is significant. While we may—as Nye routinely did—refer to the soft power of America, it is not as if the president is the commander-in-chief of all soft power. Rather, acting as an invisible hand, the soft power of America is a composite pattern contributed to by a disparate array of actors and factors: official and unofficial, public and private, intentional and unintentional. American soft power relies in large part on the success of its cultural industries (movies, music, television, etc.), and the creators of these works do not answer directly to the American state. Furthermore, the reception of cultural products may vary widely from one country to another or one group to another; to this end, Nye argues that cultural products, especially popular culture, “[do] not provide a uniform soft power resource.”^13^ This blend of direct and indirect access to soft power resources makes the projection of soft power highly complicated.^14^

But Nye’s point is not that soft power is beyond the scope of a state’s control *in toto*. He offers examples of successful strategies that America undertook during the Cold War to influence preferences in the Soviet Union. For the purposes of this article, the most significant is his focus on educational and academic exchanges, which are held up throughout the work as meaningful examples of American soft power projection. While many policy-makers feared that Soviet scientists would steal intellectual property on their research exchanges, Nye is careful to point out that “visitors vacuumed up political ideas along with scientific secrets” and “many such scientists became leading proponents of human rights and liberalization inside the Soviet Union,” offering the example of Aleksander Yakovlev.^15^ Student exchanges are presented as particularly valuable soft power projection strategies because they permit the formation of a longstanding relationship between the student and the host country. In the American case, the inclusion of over two hundred future heads of state in such exchanges is taken as a strong value proposition for future alliance-building; these students-turned-world-leaders would eventually be explicitly identified as one of the greatest assets to America’s standing on the world stage.^16^ This is not only a matter of elite diplomacy and courtship of future leaders. Nye reports that soon after 9/11, when government officials were tasked with considering public diplomacy efforts to improve America’s image in the Middle East, they quickly put forward policy proposals to increase student scholarships and visiting fellowships.^17^ Nye’s own project was largely prompted by a desire to explore how soft power might have a greater role to play in service of American foreign policy (combining hard and soft power resources to form smart power). As both an analytical tool and a strategic policy direction, soft power has since this time been discussed in many national contexts. The American emphasis present in reviewing Nye’s work is not an attempt to Americanize the Ontario case study discussed below, but instead to demonstrate the longstanding connection between education and the earliest discussions of soft power.

In addition to the broader application of soft power in a variety of policy domains,^18^ further research specifically on soft power projection through student exchanges has similarly reflected positively upon the promise of national student exchange strategies. Australia’s New Colombo Plan explicitly frames the development of student exchange programs in terms of foreign policy goals, via efforts to build relationships and project public diplomacy throughout the Indo-Pacific region.^19^ Considerations of soft power are explicitly invoked in UK student exchange programs in that cast a wide net in terms of recruitment, but also inform more targeted efforts, such as South Korea’s efforts to build lasting relationships with Uzbekistan.^20^ Japan’s use of student exchanges for soft power projection, highlighted by Nye as a significant example of the practice, has been shaped to build both regional and targeted (Japan-US) relations.^21^ Similarly, China’s development of a variety of exchange arrangements, including bilateral exchanges with the European Union, mark a significant strategic element of Chinese soft power projection.^22^ In Canada, the soft power implications of internationalization in higher education enrollments have been studied, although there is a general recognition that accelerated international student enrolments in the Canadian higher education sector is largely driven by university budgets rather than foreign policy.^23^ While these studies of the higher education sector provide interesting insights that resonate with the K-12 experience, direct attention to the dynamics of policymaking at the school board level provides an important opportunity to consider the impact of these often-overlooked actors on shaping Canadian soft power projection.

## School board budgets and international student strategies

School boards arose in the nineteenth century as education became viewed less as an aristocratic privilege and more as a public good.^24^ From the outset, this had a directly civic function, with public schools deemed to be essential in ensuring that the next generation would know and uphold the values of nineteenth-century society.^25^ Public schooling in Canadian colonies lagged behind systems in the US,^26^ and officials in Upper Canada became concerned that children sent away to American schools would return with republican commitments.^27^ To combat this, “grammar schools” were created in the province, with local authorities known as “district boards” developed to oversee them.^28^ Over the decades, school boards of different sizes were created, amalgamated, and redistributed in an effort to meet administrative and political goals of the day.^29^ In general, centralization of authority at the provincial level and standardization of curricular and funding models have diminished the autonomy of local school authorities, but the path was neither politically nor financially clean.

The roots of the current school board budgetary crisis in Ontario began with the controversial Education Quality Improvement Act of 1997. This act brought about sweeping changes to the K-12 system in Ontario, including the removal of power of local taxation from district school boards in favour of per-pupil grants determined by the Ministry of Education.^30^ While central grants had previously covered around 70 percent of educational expenditures, local taxation grew as a share of educational expenditures through the 1970s, 1980s, and 1990s.^31^ As may be expected, the power of local taxation offers greater flexibility to local decision-makers, and the centralization of taxation decisions limits school board autonomy—especially in Ontario, where boards running a deficit could have their administration taken over by Ministry officials.^32^ The per-pupil funding formula is largely made up of Grants for Student Needs, with other adjustments made for centrally identified priority areas (including rural boards, declining enrolment adjustments, and student transportation grants), with other circumstance-based revenue generation opportunities implemented locally based on central guidelines (such as educational development charges for future capital expenditures).^33^ Aside from potential withdrawals from an accumulated surplus reserves, regulatory constraints meant that local school boards were—and are—severely limited in terms of their budgetary decision-making.

Successive provincial governments in Ontario since the Education Quality Improvement Act have witnessed increased budgetary strain, due to periods of falling behind inflation as well as unfunded program expansions. While the Dalton McGuinty (2003–2013) and Kathleen Wynne (2013–2018) governments funded an inflation-adjusted 20 percent increase to per-pupil funding from 2003 to 2015, serious funding pressures remained due to changes around small class sizes, full-day kindergarten, and lingering insufficiencies for at-risk, special education, and additional-language learning programs.^34^ The subsequent Doug Ford government saw inflation-adjusted funding decline by $800 per pupil between 2017–2018 and 2021–2022.^35^ While some of the revenue decreases follow from the change to the secondary student funding that accounts for two e-learning courses (regardless of whether or not students opt out of the “optional requirement”) there is speculation that the sale of curriculum and course content may lead to new revenue streams. At present, one of the last levers solely in the hands of local school boards is fee-paying international student recruitment.

The 2015 strategy for K-12 international student exchanges frames the program as providing a richer and more global experience for students in Ontario schools, but also explicitly recognizes the attractive economic promise of international student programs. The four stated goals of the strategy are: the increased interaction of Ontarian students with the world through meeting international students, studying international languages, and twinning with other schools; high-quality experiences for international students in Ontario, both in terms of curricular and extracurricular experiences; showcasing and developing Ontario’s expertise in education; and providing international students with pathways to post-secondary education and the workforce.^36^ In its more fiscally oriented pages, however, the strategy notes that “some boards may also be able to offset declining enrolment through accepting larger numbers of tuition-based visa students, gaining new revenue sources in the process.”^37^ The school board benefits in different ways from domestic and international students. As the strategy notes, international students “choose Ontario as a place to learn and to share intercultural experiences,” and while they are a valuable addition to the learning environments that they join; however, the report notes that “they may also be recruited by boards in response to issues of declining enrolment in some areas, or as an additional source of revenue”—a much more pragmatic rationale.^38^

The years since the strategy’s publication have seen increases to the international student program, with readily apparent motivations and difficulties.^39^ Reports in 2017 spoke of rapidly growing programs across the province, with recruiters attending international events and drawing on global networks.^40^ With rapid expansion, however, cracks began to show. Investigative reporters found an unregulated industry where students in their early teens were sometimes left stranded by insufficiently attentive host families or custodians.^41^ Scholars have highlighted that the attractiveness of the revenue stream, given the centralization and tightening of purse strings for education, have led to a financialized focus of policy development—creating a feedback loop where increased budgetary pressures lead to expanded recruitment to boost revenues, only to justify a slowing of provincial investment in education.^42^ While international student programs offer a revenue stream that is within the school board’s control, the funding gaps that have only deepened since the late 1990s have given school boards a strong financial incentive to build their own strategies. The majority of media and political attention around international student recruitment has focused on the post-secondary level, which involves a much larger volume of students. However, as I argue in the following section, recalling the role of education in soft power means that ignoring the complex issue of K-12 international students also overlooks the potential political implications of school board actions on the international stage.

## School boards and soft power paradiplomacy

To suggest that school boards are accidental paradiplomats projecting Canada’s soft power is to say that they have stumbled into the business of building cultural bridges to export understandings of Canadian society. This is not, as in the case of Quebec, a purposeful paradiplomacy whereby a subnational government seeks to articulate a strong message about its culture, identity, and society, and to forge beneficial networks on the international stage. Instead, financial pressures have left school boards with international student recruitment as one of the only methods of revenue generation within their control. Yes, the provincial-level strategy highlights that domestic students will benefit from the diversity of experiences that such programs may bring to their learning environments, but in a post-amalgamation Ontario education sector where school boards now service many communities, the primary benefit at the school board level of analysis is financial. And yet, I argue that there remains a force of soft power projection that accompanies the programming. This section proceeds with an analysis of promotional materials and program structures alongside the logic of education-based soft power projection described by Nye.

While many individual school boards have developed specialized program offerings, the standard international student experience involves a one-year exchange. At the elementary level, this typically involves a parent or guardian moving to the school community to live with the child, and at the secondary school level, students are generally placed with a “home-stay” family that has been vetted by the school board. The Ontario Association of School Districts International, the organization representing school boards that participate in international student exchange programs, explicitly links the discussion of typical experiences of international students to cultural exposure:

Many students stay for several years during high school with an aim of pursuing a post-secondary pathway at a Canadian university or college; other students study in Ontario for a shorter time period with the goal of improving language skills and learning about a different culture…

Each long-term student is required to have a legal custodian while studying in Ontario, and while some students live with a relative or family friend, most students live with a family in Homestay. Living with an Ontario family for an extended time period is an excellent way for international students to learn about Canadian culture in a caring and supportive environment.^43^

The opportunity to learn about Canadian culture, including what OASDI calls “home culture,” is presented as part of the value proposition to potential students. Frequently, program portals on school board websites include details on field trips and excursions designed to further explore Canadian cultural sites. School boards in the Ottawa area note trips to visit the Parliament buildings, the Byward Market, CN Tower, Niagara Falls, Kingston, and Montreal, and to attend a local hockey game.^44^ Toronto District School Board, the largest school board in the province, also offers a dedicated cultural immersion program for group-based exchanges, which include many of the same elements, including language training, immersion in local schools, placement with home-stay families, friendships with domestic students, and visits to sites of cultural significance.^45^ Despite the incentive structure for school boards tilting heavily towards the financial aspects of the program, both promotional materials and program structures indicate an awareness that cultural exposure, immersion, and experience form key elements of the value proposition of international student experiences.

Writing for leaders in the American higher education sector, Nye offers a useful framework for considering how educational programming can serve as soft power projection. Even if the case of international student programs designed by Ontario school boards are accidentally paradiplomatic, considering their entanglement with soft power projection provides an interesting window into the current status of Canadian soft power. Nye suggests that the attractiveness of a nation’s culture is the first of three key resources of soft power, and notes that educational and academic exchanges are important opportunities because of their immersive experience.^46^ Although high tuition fees may appear at first glance to be exclusionary and potentially a limiting factor for soft power projection, this actually falls broadly in line with Nye’s understanding of such educational exchanges as being an opportunity for foreign elites.^47^ This economic elitism is perhaps even a strength of the program from Nye’s perspective: “because exchanges affect elites, one or two key contacts may have a major political effect.”^48^ The efforts by school boards to offer opportunities to engage with cultural highlights alongside their educational programming seeks to present national culture in the most favourable terms, foregrounding the attractiveness of Canada. Here, too, we find a symbiosis between the development of a clear value proposition—with competition for international student recruits, school boards want to highlight the quality of cultural experiences and excursions that complement the academic elements—and Canadian soft power projection. As Nye notes, a culture’s level of attractiveness is an important factor in building the relationships that will subsequently serve as a soft power resource for a country.^49^ Again, we find an alignment of interests between school boards, seeking to close financial gaps, and national soft power politics.

This accidental alignment of interests informs my suggestion that school boards are accidental paradiplomats. The bleak financial situation of Ontario school boards has left these institutions with few options to explore as they attempt to meet the needs of their students and represent constituents’ interests. In an effort to recruit international students, they have recognized that developing attractive cultural experiences can improve the quality of their international education programs. Stronger programs promise to benefit international students (as improved programs may better meet their needs), domestic students (offering an opportunity for richer and more diverse classrooms with global perspectives, as well as increasing the funding for schools), and the boards’ bottom lines. But at the same time, there are impacts beyond the school system that relate to the country’s soft power projection. By designing programs that amplify the attractiveness of Canadian culture to elite members of different nations, the school boards are developing further soft power resources in line with Nye’s prescriptions of soft power. That the leadership on this effort comes from the oft-ignored local level of school board governance is particularly important, as it demonstrates that the analysis of soft power projection must be analyzed in terms of both intentional and unintentional policy efforts, in expected and unexpected places.

## Conclusion

Following then minister of foreign affairs Lloyd Axworthy’s explicit endorsement of soft power in the 1990s, a debate erupted among the Canadian foreign policy commentariat.^50^ Often critical of the explicit branding of the softer touch that Axworthy advocated,^51^ this debate focused largely on the strategic direction of the government. The timing of the shift towards soft power policy conveniently fit with budget cuts towards hard power resources during the Jean Chrétien government—a point that was not lost on critics.^52^ Although there may be great value gained from paying attention to the federal government’s direct efforts to project soft power,^53^ this article has argued for a much wider net to be cast to appreciate the broad network of actors contributing in multiple and often uncoordinated ways to Canadian soft power projection. Just as Hollywood contributes to the projection of American culture without coordination with the American foreign policy establishment, so too do actors beyond the Canadian federal government’s umbrella. Further research is needed to understand the impact of non-federal and, more broadly, non-state instances of soft power projection in Canada.^54^

A second conclusion that I draw from this discussion speaks to the fields Canadian political science and International Relations. Ontario school boards have received relatively limited scholarly attention, owing in part to their constraints around policy-related and financial decision-making. However, the case described above demonstrates that politically relevant externalities may follow from the actions of these oft-overlooked political bodies. Recent research by Jennifer Wallner and Stéphanie Chouinard on political identity-construction through history curricula in Ontario and Quebec demonstrates the importance of taking seriously the broader political implications of educational policy.^55^ Even in situations where decisions around funding, curriculum, and program construction are made at a higher level of government, local implementation plans and resource decisions may introduce new elements for consideration.

It might have been easy to explain away the lack of attention if educational exchanges were not known to be powerful techniques of soft power promotion. Similarly, if public education systems in Canada had no historical or contemporary connection to civic education or political notions of citizenship or national identity, we might accept this case as an unforeseeable accident. Yet neither is the case. It may well be the case that financial prospects are motivating school board actions in expanding international student recruitment efforts, yet the political role of Canadian public education and the established connection between educational exchanges and soft power projection mean that the significance of the accidental paradiplomacy should be self-evident. Further attention must be paid to this issue to assess the soft power ramifications of these budgetary decisions and to adjudicate if soft power and its implications demands more consideration. This should be more than a matter of marketing materials promising a trip to the CN tower or to taste maple syrup—the question of what culture is shared is one of utmost importance to understand the radically unknown content of these programs.

## Notes

1. Joseph S. Nye, *Soft Power: The Means to Success in World Politics* (New York: Public Affairs, 2004), 99.

2. See, for example, Pierre Bourdieu and Jean-Claude Passeron, *Reproduction in Education, Society and Culture,* 2^nd^ ed. (London, Thousand Oaks, and New Delhi: Sage, 1990).

3. Nye, *Soft Power*, 124; Carol Atkinson, “Does soft power matter? A comparative analysis of student exchange programs 1980–2006,” *Foreign Policy Analysis* 6, no. 1 (2010): 1–22.

4. Sachin Maharaj, “From oversight to advocacy: An examination of school-board leadership,” *Leadership and Policy in Schools* 19, no. 3 (2020): 431–443; R. Michael McGregor and Jack Lucas, “Who has school spirit? Explaining voter participation in school board elections,” *Canadian Journal of Political Science/Revue canadienne de science politique* 52, no. 4 (2019): 923–936; Anthony Piscitelli and Andrea Perrella, “Stealth democracy and the school board trustee,” *Canadian Public Administration* (2022); Paul W. Bennett, *The State of the System: A Reality Check on Canada's Schools* (Montréal and Kingston: McGill-Queen's University Press, 2020); Jack Lucas, *Fields of Authority: Special Purpose Governance in Ontario, 1815–2015* (Toronto: University of Toronto Press, 2016).

5. Alexander S. Kuznetsov, *Theory and Practice of Paradiplomacy: Subnational Governments in International Affairs* (London and New York: Routledge, 2014); Rodrigo Tavares, *Paradiplomacy: Cities and States as Global Players* (Oxford: Oxford University Press, 2016); Peter Jones, *Track Two Diplomacy in Theory and Practice* (Stanford: Stanford University Press, 2015).

6. Andre Lecours, “Paradiplomacy: Reflections on the foreign policy and international relations of regions,” *International Negotiation* 7, no. 1 (2002): 91–114; James T. McHugh, “Paradiplomacy, protodiplomacy and the foreign policy aspirations of Quebec and other Canadian provinces,” *Canadian Foreign Policy Journal* 21, no. 3 (2015): 238–256; Stéphane Paquin, “Identity paradiplomacy in Québec,” *Quebec Studies* (2018); Stéphane Paquin, X. Hubert Rioux, David Eiser, Graeme Roy, and Ian Wooton, “Quebec, Scotland, and substate governments’ roles in Canadian and British trade policy: Lessons to be learned,” *International Journal* 76, no. 1 (2021): 85–105.

7. David Dyment, “The Ontario government as an international actor,” *Regional & Federal Studies* 11, no. 1 (2001): 55–79; Charles-Louis Labrecque and Scott Harrison, “Canadian provinces and foreign policy in Asia,” *International Journal* 73, no. 3 (2018): 429–448; Patrick J. Smith, “The making of a global city: Fifty years of constituent diplomacy–the case of Vancouver.” *Canadian Journal of Urban Research* 1, no. 1 (1992): 90–112; Richard Stren and Abigail Friendly, “Toronto and São Paulo: Cities and international diplomacy,” *Urban Affairs Review* 55, no. 2 (2019): 375–404; Caroline Dunton and Veronica Kitchen, “Paradiplomatic policing and relocating Canadian foreign policy,” *International Journal* 69, no. 2 (2014): 183–197.

8. Leah Sarson, “’You cannot trade what is not yours’: Indigenous governance and the NAFTA negotiations,” *American Review of Canadian Studies* 49, no. 2 (2019): 332–347. Sarson notes resonances of Indigenous paradiplomacy in theories of multilevel governance, for example, in Christopher Alcantara and Jen Nelles, *A Quiet Evolution: The Emergence of Indigenous-Local Intergovernmental Partnerships in Canada* (Toronto: University of Toronto Press, 2016); Kiera L. Ladner, “Colonialism isn't the only answer: Indigenous Peoples and multilevel governance in Canada,” in Marian Sawer and Melissa Haussman, eds., *Federalism, Feminism and Multilevel Governance* (London and New York: Routledge, 2016), 67–82. Martin Papillon, “Adapting federalism: Indigenous multilevel governance in Canada and the United States,” *Publius: The Journal of Federalism* 42, no. 2 (2012): 289–312.

9. Joseph Nye, *Bound to Lead: The Changing Nature of American Power* (New York: Basic Books, 1990), 188, 192; Nye, *The Paradox of American Power: Why the World’s Only Superpower Can’t Go It Alone* (Oxford and New York: Oxford University Press, 2002), xi–xv.

10. Nye, *The Paradox of American Power*, 9.

11. Because of this commitment to the original sources, this means that Nye’s American-centrism carries through part of this section.

12. Nye, *Soft Power*, 7.

13. Ibid., 52.

14. Ibid., 99.

15. Nye, *Soft Power,* 45; Joseph Nye, “Soft power and higher education,” *Forum for the Future of Higher Education (Archives)* (Boulder: EDUCAUSE, 2005), 11–14.

16. Nye, *Soft Power,* 109–110, 44.

17. Ibid., 121. Nye also reports that European nations recognized the value of student exchanges as a means by which a country’s image might be improved, leading to concerted efforts to increase student recruitment. See Nye, *Soft Power*, 82.

18. Ibrahim Kalin, “Soft power and public diplomacy in Turkey,” *Perceptions: Journal of International Affairs* 16, no. 3 (2011): 5–23; Seow Ting Lee, “Vaccine diplomacy: Nation branding and China’s COVID-19 soft power play,” *Place Branding and Public Diplomacy* (2021): 1–15; Jongryn Mo, “South Korea’s middle power diplomacy: A case of growing compatibility between regional and global roles,” *International Journal* 71, no. 4 (2016): 587–607; Yiwei Wang, “Public diplomacy and the rise of Chinese soft power,” *The Annals of the American Academy of Political and Social Science* 616, no. 1 (2008): 257–273.

19. Caitlin Byrne, “Australia’s New Colombo plan: Enhancing regional soft power through student mobility.” *International Journal* 71, no. 1 (2016): 107–128; Natalie Laifer and Nicholas Kitchen, “Making soft power work: Theory and practice in Australia’s international education policy.” *Politics & Policy* 45, no. 5 (2017): 813–840.

20. Sylvie Lomer, “Soft power as a policy rationale for international education in the UK: A critical analysis,” *Higher Education* 74, no. 4 (2017): 581–598; Eriks Varpahovskis, “Education as a soft power tool: Korea’s approach toward Uzbekistan,” in Kadir Jun Ayhan, ed., *Korea’s Soft Power and Public Diplomacy (Seoul: Hangang Network,* 2017), 101–123.

21. Nye, *Soft Power*, 110; Nissim Kadosh Otmazgin, “Geopolitics and soft power: Japan’s cultural policy and cultural diplomacy in Asia,” *Asia-Pacific Review* 19, no. 1 (2012): 37–61; Ellen Mashiko and Horie Miki, “Nurturing soft power: The impact of Japanese-US university exchanges,” In Yasushi Watanabe, Yasushi Watanabe, and David L. McConnell, eds., *Soft Power Superpowers* (London and New York: Routledge, 2015), 107–128.

22. Natalie Yan Hong, “EU-China education diplomacy: An effective soft power strategy?” *European Foreign Affairs Review* 19, no. Special (2014); Jian Li and Eryong Xue, “The rising soft power’: An educational foreign exchange and cooperation policy conceptual framework in China,” *Educational Philosophy and Theory* (2022): 1–10.

23. Roopa Desai Trilokekar, “International education as soft power? The contributions and challenges of Canadian foreign policy to the internationalization of higher education,” *Higher Education* 59 (2010): 131–147; Trilokekar, “From soft power to economic diplomacy? A comparison of the changing rationales and roles of the US and Canadian federal governments in international education,” Research & Occasional Paper Series, CSHE, 2.15. Center for Studies in Higher Education, University of California-Berkeley, 2015; Merli Tamtik, “Who governs the internationalization of higher education? A comparative analysis of macro-regional policies in Canada and the European Union,” *Comparative and International Education* 46, no. 1 (2017); Sharon Stein, “National exceptionalism in the ‘EduCanada’ brand: Unpacking the ethics of internationalization marketing in Canada,” *Discourse: Studies in the Cultural Politics of Education* 39, no. 3 (2018): 461–477; Amira El Masri Trilokekar and Hani El Masry, “Power, politics, and education: Canadian universities and international education in an era of new geopolitics,” *Canadian Journal of Higher Education* 50, no. 3 (2020): 79–95; Rebeca Heringer, “From enrolment rates to collaborative knowledge production: A critique to the internationalization of higher education in Canada.” *Higher Education for the Future* 7, no. 2 (2020): 169–186.

24. Jennifer Wallner, *Learning to School: Federalism and Public Schooling in Canada* (Toronto: University of Toronto Press, 2014).

25. Carmen Neilson reminds us that this is not necessarily a beneficial endeavour: “Schooling was essential to the process of moral regulation, whereby capitalism, liberalism, patriarchy, and white supremacy were normalized.” See Nielson, *Private Women and the Public Good: Charity and State Formation in Hamilton, Ontario, 1846–93* (Vancouver: UBC Press, 2014), 19. See also Bruce Curtis, *True Government by Choice Men? Inspection, Education, and State Formation in Canada West*, no. 17 (Toronto: University of Toronto Press, 1992).

26. Bruce Curtis, *Ruling by Schooling Quebec: Conquest to Liberal Governmentality: A Historical Sociology* (Toronto: University of Toronto Press, 2012), 7.

27. Lucas, *Fields of Authority*, 107.

28. Ibid.

29. For a federal perspective, see Wallner, *Learning to School*. For an Ontarian perspective, see Lucas, *Fields of Authority*. For the linguistic and cultural politics of Quebec, see Curtis*, Ruling by Schooling*.

30. Ontario English Catholic Teachers' Assn. v. Ontario (Attorney General) 2001 SCC 15.

31. George J. Bedard and Stephen B. Lawion, “The struggle for power and control: Shifting policy-making models and the Harris agenda for education in Ontario,” *Canadian Public Administration* 43, no. 3 (2000): 241–269.

32. Jennifer Wallner, *Learning to School: Federalism and Public Schooling in Canada* (Toronto: University of Toronto Press, 2014), 62–63; Ontario English Catholic Teachers' Assn. v. Ontario (Attorney General) 2001 SCC 15.

33. Ontario Ministry of Education, “*2023–24 grants for student needs funding*,” 2023:B04, Toronto, 17 April 2023, https://efis.fma.csc.gov.on.ca/faab/Memos/B2023/B04_EN.pdf (accessed 3 January 2024); Ontario Ministry of Education, *Education Development Charges Guidelines* (Toronto: Queen’s Printer for Ontario, 2002). https://efis.fma.csc.gov.on.ca/faab/Capital%20Programs%20Branch/EDC%20Guideline.pdf

34. Hugh Mackenzie, *Harris-Era Hangovers: Toronto School Trustees’ Inherited Funding Shortfall* (Toronto: Canadian Centre for Policy Alternatives, 2015).

35. Ricardo Tranjan, “The straight answer: Education funding in Ontario has dropped since 2018,” The Monitor, Canadian Centre for Policy Alternatives, Ottawa, 27 April 2022, https://monitormag.ca/articles/the-straight-answer-education-funding-in-ontario-has-dropped-since-2018 (accessed 26 December 2023).

36. Ontario Ministry of Education (EDU), *Ontario’s Strategy for K-12 International Education* (Toronto: Queen’s Printer for Ontario, 2015), 20*ff*.

37. Ibid., 17.

38. EDU, *Ontario’s Strategy,* 10

39. These are reported in terms relative to K-12 recruitment, not in comparison to post-secondary international student recruitment, which far exceeds the K-12 programs.

40. Paolo Loriggio, “Canadian high schools welcoming growing number of international students, boards say,” *Toronto Star*, 30 August 2017, https://www.thestar.com/news/canada/canadian-high-schools-welcoming-growing-number-of-international-students-boards-say/article_93dbd444-23cc-5f65-afc0-e57d9c4a6c96.html (accessed 26 December 2023).

41. Lisa Xing and Lu Zhou, “'The system is full of holes': Experts say young foreign students left vulnerable by unregulated industry,” CBC News, 22 February 2018, https://www.cbc.ca/news/canada/toronto/underage-visa-students-falling-through-cracks-in-canada-1.4525664 (accessed 26 December 2023).

42. Lana Parker, Bonnie Stewart, and Natalie Delia Deckard, “Canada’s high schools are underfunded and turning to international tuition to help,” *The Conversation Canada*, 27 February 2020, https://theconversation.com/canadas-high-schools-are-underfunded-and-turning-to-international-tuition-to-help-127753 (accessed 26 December 2023).

43. Ontario Association of School Districts International (OASDI), “Long-term programs for international students in Ontario,” Richmond Hill, 2023, https://www.oasdi.ca/international-education/programs/long-term/ (accessed 8 June 2023).

44. Ottawa-Carleton Education Network, “OCENT rebounds,” Ottawa, 2022, https://ocenet.ocdsb.ca/news/newsletter-fall-2022/ocenet_rebounds (accessed 8 June 2023); Ottawa Catholic School Board, “International Education Week: The positive impact of international education,” Ottawa, 16 November 2021, https://international.ocsb.ca/news/international-education-week-the-positive-impact-of-international-education/ (accessed 26 December 2023).

45. Toronto District School Board, “TDSB cultural immersion program,” 2023, https://www.tdsb.on.ca/Portals/0/docs/TDSB_CulturalImmersionProgram.pdf

46. Nye, “Soft power and higher education.”

47. Unlike at the post-secondary level, where high tuition fees can be understood as a cost of the pathway to citizenship, the shorter duration of K-12 exchanges makes the program designed more for experiential than citizenship-related purposes. This is not, however, to say that citizenship desires play no part, either in the decision to participate in the first place or in the enjoyment of the Canadian experience inspiring a future application for citizenship.

48. Nye, “Soft power and higher education,” 14.

49. Nye, *Soft Power*, 45 and *passim*.

50. Lloyd Axworthy, “Canada and human security: The need for leadership,” *International Journal* 52, no. 2 (1997): 183–196; Axworthy, “Why soft power is the right policy for Canada,” *Ottawa Citizen,* 25 April 1998, B6.

51. Kim Richard Nossal, “Foreign policy for wimps,” *Ottawa Citizen,* 23 April 1998, A19; and Fen Hampson and Oliver Dean, “Pulpit diplomacy: A critical assessment of the Axworthy doctrine,” *International Journal* 53, no. 3 (1998): 379–406.

52. Kim Richard Nossal, Stéphane Roussel, and Stéphane Paquin, *The Politics of Canadian Foreign Policy* (Kingston: Queen’s School of Policy Studies/McGill-Queen’s University Press, 2015), 80–81.

53. Evan H. Potter, *Branding Canada: Projecting Canada's Soft Power through Public Diplomacy* (Montréal and Kingston: McGill-Queen's University Press, 2008).

54. To offer one example, we might consider the cultural impact of the television program *Schitt’s Creek,* which was generally regarded as a representing distinctly—if subtly—Canadian culture. See Victoria Kannen, “Loving and loathing on Schitt’s Creek: How representations of emotion, identities, and nation matter,” Victoria Kannen and Neil Shyminsky, eds., *The Spaces and Places of Canadian Popular Culture* (Toronto: Canadian Scholars Press, 2019), 56–66; Margaret Lyons, “Schitt’s Creek brings love, Canadian style,” *The New York Times*, 15 January 2019, https://www.nytimes.com/2019/01/15/arts/television/schitts-creek-season-5.html (accessed 26 December 2023);

55. Jennifer Wallner and Stéphanie Chouinard, “Nation-building and curriculum in Canada,” in Daniel Tröhler, ed., *Education, Curriculum and Nation-Building* (London and New York: Routledge, 2023), 149–172; Stéphanie Chouinard and Jennifer Wallner, “What history, for what nation? The narrative framework of French- and English-language history curricula in Ontario from a comparative perspective,” Paper presented at the Canadian Political Science Association 2024 annual conference.

